# Synergistic increase in anti-cancer stemness activity and pro-apoptotic effects in human glioblastoma cancer stem-like cells by combination treatment with temozolomide and corosolic acid

**DOI:** 10.7150/ijms.122961

**Published:** 2025-10-20

**Authors:** Chuan-Yi Kao, Hsiang-Yao Shih, Yi-Hsien Hsieh, Chung-Jung Liu, Ming-Chun Hung, Jeng-Yih Wu, Yi-Chen Lin, Chien-Min Chen

**Affiliations:** 1 Institute of Medicine, Chung Shan Medical University, Taichung, Taiwan.; 2 Department of Psychiatry, Chung Shan Medical University Hospital, Taichung, Taiwan.; 3 Division of Gastroenterology, Department of Internal Medicine, Kaohsiung Medical University Hospital, Kaohsiung, Taiwan.; 4 Department of Medical Research, Chung Shan Medical University Hospital, Taichung, Taiwan.; 5 Regenerative Medicine and Cell Therapy Research Center, Kaohsiung Medical University, Kaohsiung, Taiwan.; 6 Department of Medicine, Faculty of Medicine, College of Medicine, Kaohsiung Medical University, Kaohsiung, Taiwan.; 7 Division of Neurosurgery, Department of Surgery, Changhua Christian Hospital, Changhua, Taiwan.; 8 Department of Biomedical Sciences, National Chung Cheng University, Chiayi, Taiwan.

**Keywords:** Temozolomide, Corosolic Acid, Glioblastoma, Cancer stem cells, Apoptosis, Migration, Invasion

## Abstract

Glioblastoma (GB) is a highly aggressive brain cancer with poor prognosis and a five-year survival rate of only 4-5%, largely due to challenges in surgical removal and radiotherapy limitations. Corosolic acid (CA), a natural pentacyclic triterpene, exhibits promising anti-cancer activity against GB. In this study, we founded that GBM8401-derived cancer stem cells (GBM8401-CSCs) display increased stemness, proliferation, migration, invasion, and elevated cancer stemness factors (Nestin, OCT4, CD133) compared to parental cells. CA treatment dose-dependently inhibited these malignant features and downregulated key cancer stemness genes. Combined with Temozolomide (TMZ), CA synergistically suppressed GBM8401-CSCs growth, colony formation, migration, invasion, and promoted apoptosis more effectively than either CA or TMZ alone and significantly reduced sphere formation and cancer stemness gene expression. Molecular docking results show a strong affinity of TMZ and CA for CD133 and OCT4 proteins, highlighting distinct molecular interactions. These results suggest that CA, especially in combination with TMZ, holds therapeutic potential for targeting human GB-CSCs.

## Introduction

Glioblastoma (GB) is the most common and aggressive primary brain tumor in adults, classified as a World Health Organization (WHO) grade IV astrocytoma 1, 2. Accounting for 15% of brain tumors and 3-4% of cancer-related deaths, GB represents the most malignant form of glioma 3. Its highly invasive nature allows tumor cells to infiltrate surrounding brain tissue, rendering complete surgical resection nearly impossible and leading to frequent recurrence 4. Despite advances in multimodal therapies including maximal safe resection, radiotherapy, and temozolomide (TMZ) chemotherapy, GB remains a devastating diagnosis. Median survival is 14-15 months with treatment, while fewer than 5% of patients survive beyond five years 5, 6. The minimal improvement in long-term survival over the past three decades underscores the urgent need for novel treatment strategies 7, 8. A deeper understanding of the molecular mechanisms driving GB progression, invasion, and therapeutic resistance is essential.

Glioblastoma cancer stem cells (GB-CSCs), also referred to as glioblastoma stem-like cells (GSCs), play a pivotal role in tumor initiation, progression, recurrence, and resistance to therapy. These cells possess key stem cell-like properties, including the ability to self-renew, differentiate into multiple neural lineages, and withstand conventional treatments, which collectively contribute to the highly aggressive nature of glioblastoma 9. GB-CSCs share many features with normal neural stem cells (NSCs), including the expression of specific markers such as OCT4, CD133 and Nestin 10. A defining and clinically challenging feature of GB-CSCs is their marked resistance to chemotherapy and radiotherapy 11. Therefore, targeting GB-CSCs represents a promising therapeutic strategy for improving GB treatment outcomes.

Corosolic acid (CA), a ursane-type pentacyclic triterpenic acid, is a natural compound widely distributed in plants such as loquat leaves 12 and guava 13. CA has shown promising anti-cancer potential through a variety of mechanisms, positioning it as a candidate for cancer therapy 14, 15. *In vitro* studies have shown that CA can inhibit GB cell viability, induce apoptosis, and modulate signaling pathways involved in tumor progression, such as NF-κB 16, STAT3 17 and GAS6/AXL/JAK Axis 18. These pathways are frequently dysregulated in GB, indicating a possible mechanism through which CA may exert therapeutic effects. Studies investigating other natural compounds, such as curcumin 19 and resveratrol 20, have shown similar mechanisms and synergistic effects with TMZ in GB models, providing a comparative framework for CA-based approaches.

This study aimed to evaluate the anti-tumor effects of corosolic acid (CA), both alone and in combination with the chemotherapeutic agent temozolomide (TMZ) in human GB-CSCs. Specifically, the investigation focused on cell proliferation, migration, invasion and apoptosis. We hypothesized that CA could effectively suppress the malignant properties of human GB-CSCs and act synergistically with TMZ to enhance its therapeutic efficacy, thereby addressing the challenges of GB-CSCs recurrence and drug resistance.

## Materials and Methods

### Reagents

Corosolic acid (CA; CFN98685) was purchased from ChemFaces company (Wuhan, Hubei, China). Temodal® Capsules (TMZ; 100 mg) was provided from Orion Corporation (Turku, Finland). Bovine serum albumin (BSA), epidermal growth factor (EGF) (10 ng/mL), recombinant human fibroblast growth factor (bFGF) (10 ng/mL), and insulin (5 μg/mL) were obtained from R&D Systems (Minneapolis, MN, USA). Cell culture reagents including isopropanol, 3-(4,5-Dimethylthiazol-2-yl)-2,5-diphenyltetrazolium bromide (MTT), dimethyl sulfoxide (DMSO), sodium chloride (NaCl), sodium dodecyl sulfate (SDS), Tris-HCl, and trypsin/EDTA were purchased from Sigma-Aldrich (St. Louis, MO, USA). GoScript Reverse Transcription Mix kit and GoTaq qPCR Master Mix were purchased from Promega Corporation (Madison, WI, USA). Matrigel was purchased from BD Biosciences (San Jose, CA, USA).

### Cell culture and drug treatment condition

GBM8401-CSCs were seeded into 6-cm culture dishes (5 × 10^3^ cells/ well) and cultured for 24 h in RPMI 1640 medium containing epidermal growth factor (EGF; 10 ng/mL), recombinant human fibroblast growth factor (bFGF; 10 ng/mL), and insulin (5 μg/mL).

### Evaluation of cell growth and proliferation

GBM8401-CSCs were seeded into 6-cm culture dishes (5 × 10^3^ cells/well) and incubated for 24 h. Cells were treated with various concentration of CA (10, 15, 20 μM) for an additional 24 h. For the combination treatment assay, cells were treated with CA (15 μM), TMZ (600 μM), or a combination of CA (15 μM) and TMZ (600 μM) and incubated for 24 h. The cell growth rate was evaluated using MTT reagent (0.5 mg/mL). After treatment, the cells were washed twice with PBS. Subsequent procedures were performed as previously described 21. The absorbance at 570 nM was determined using a Multiskan MS ELISA reader (Labsystems, Helsinki, Finland).

### Annexin V/PI double staining assay of apoptosis

Apoptosis induction was evaluated using the Muse Annexin V and Dead Cell Assay Kit (BD Biosciences, San Jose, CA, USA)**.** Briefly, GBM8401 CSC-like cells were incubated with CA, TMZ, or a combination of CA and TMZ for 24 h. Cells then were resuspended in Annexin V and Dead Cell Assay Kit solution and incubated for 15 mins. Stained cells (1 × 10^4^) were collected and analyzed using a Muse Cell Analyzer (EMD Millipore, Billerica, MA, USA).

### Clinical human glioma database

The mRNA expression levels of Nestin, OCT4, and CD133 in normal brain (N = 207) and GB (T = 163) was determined using data in the GEPIA2.0 database (http://gepia.cancer-pku.cn).

### Migration and invasion assay

Cell migration and invasion were assayed following the method described 22. GBM8401-CSC cells were treated with the indicated drugs for 24 h and then seeded (5 × 10^5^ cells/chamber) into serum-free medium in non-Matrigel chambers (for migration) or Matrigel-membrane-coated chambers (for invasion). The lower chamber contained RMPI1640/10% FBS medium. After treatment for 24 h, the membranes were fixed with methanol and stained with crystal violet. The number of migrated cells was counted using a light microscope in four fields for each treatment well.

### RT-qPCR assay

The GoScript Reverse Transcription Mix was used for reverse transcription of mRNA extracted from GBM8401 CSC-like cells treated with CA, TMZ, or a combination of CA and TMZ. For qPCR assay, the GoTaq qPCR Master Mix was used with the human-specific primer sequences as listed, Nestin forward: 5'-CCTCAGCTTTCAGGACCCCAAG-3'; Nestin reverse: 5'-CACAGGTGTCTCAAGGGTAGG-3'; OCT4 forward: 5'-CCTGAAGCAGAAGAGGATCACC-3'; OCT4 reverse: 5'- AAAGCGGCAGATGGTCGTTTGG-3'; CD133 forward: 5'- CGTGATTTTTTACTACCTGGGCTTA-3'; CD133 reverse: 5'-AGCCTCGGGTGGTCGG-3'; GAPDH forward: 5'- CATCATCCCTGCCTCTACTG-3'; GAPDH reverse: 5'-GCCTGCTTCACCACCTTC-3'. The qPCR cycling conditions were as follows: 95 °C for 2 min, 40 cycles at 94 °C for 15 s, and 60 °C for 60 s (StepOnePlus real-time PCR machine). Relative mRNA expression was normalized to GAPDH and calculated using the 2^-ΔΔCT^ method.

### Molecular docking

The predicted three-dimensional structures of human CD133 (PROM1; UniProt ID: O43490; model ID: AF-O43490-F1-model_v4) and OCT4 (POU5F1; UniProt ID: Q01860; model ID: AF-Q01860-F1-model_v4_OCT4) were obtained from the AlphaFold Protein Structure Database. Prior to molecular docking, the protein structures were prepared using PyRx (version 0.8). The small molecules corosolic acid (CA, PubChem CID: 6918774) and temozolomide (TMZ, PubChem CID: 5394) were retrieved from the PubChem database in SDF format. The ligands were energy-minimized using the Universal Force Field (UFF) in Open Babel. A total of nine binding poses were generated and ranked based on their predicted binding affinities (kcal/mol). The top-ranked pose with the lowest binding energy was selected for further analysis. Protein-ligand interactions were visualized and analyzed using Discovery Studio 2025 Client.

### Statistical analysis

Data are presented as the mean ± standard error of the mean (SEM). Data were analyzed using GraphPad Prism. Student's t-test and one-way analysis of variance (ANOVA) were performed using GraphPad Prism. p < 0.05 was considered statistically significant.

## Results

### Cancer stemness, proliferation, and metastatic characteristics of GBM8401-CSCs

**Figure 1:**
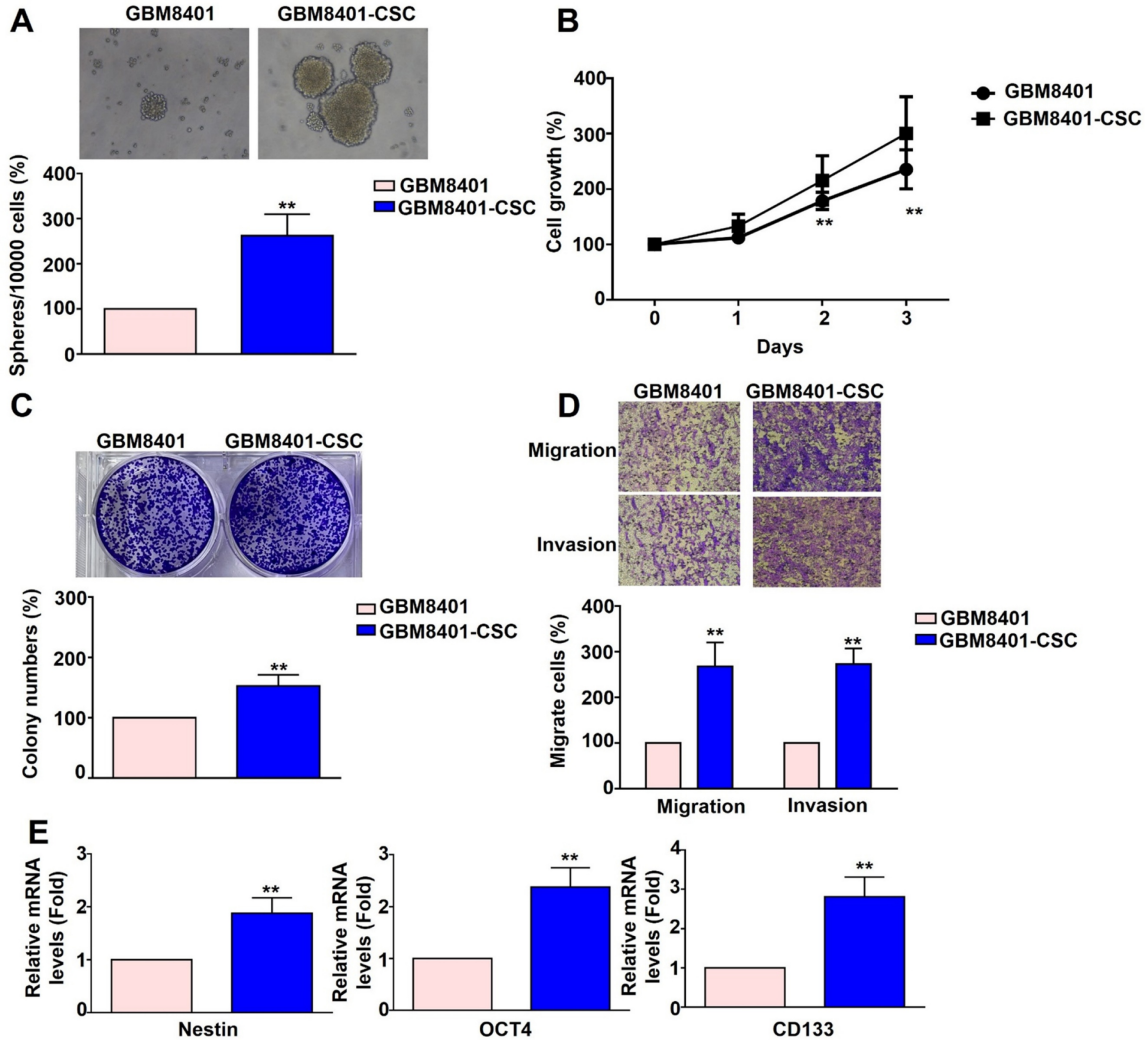
** Comparison of the characteristics of GBM8401 cells and GBM8401-CSCs across multiple aspects.** (A) Human GBM8401 cells and GBM8401-CSCs were seeded at a density of 10,000 cells and cultured for five days, after which they were harvested to observe 3D sphere formation. (B) Cell growth of GBM8401 cells and GBM8401-CSCs was analyzed on days 0, 1, 2, and 3 using the MTT assay. (C) The colony formation ability of GBM8401 cells and GBM8401-CSCs was assessed by seeding cells at appropriate dilutions and allowing colonies to form over a two-week period. (D) Migration and invasion assays were conducted to assess the metastatic potential of GBM8401 cells and GBM8401-CSCs. (E) Stem cell marker expression in GBM8401 cells and GBM8401-CSCs was evaluated by analyzing mRNA levels of Nestin, OCT4, and CD133. **, *p*<0.01 *versus* GBM8401 indicates a significant difference.

Human GBM8401 cells and GBM8401-CSCs were seeded at a density of 10,000 cells and cultured for five days. Following incubation, the cells were harvested to assess 3D sphere formation. GBM8401-CSC formed more neurospheres (Figure 1A), had stronger colony-forming ability (Figure 1C), and showed higher mRNA expression of cancer stemness factors than did GBM8401 cells (Nestin, OCT4, CD133) (Figure 1E). The proliferation of GBM8401 cells and GBM8401-CSCs was assessed on days 0, 1, 2, and 3 using the MTT assay. The growth rate of GBM8401-CSC *in vitro* was greater than that of GBM8401 cells (Figure 1B). The metastatic potential of GBM8401 cells and GBM8401-CSCs was then evaluated using migration and invasion assays. GBM8401-CSC showed significantly higher migration and invasion ability (Figure 1D) than did than did GBM8401 cells. These results show that compared to GBM8401 cells, GBM8401-CSC had stronger stem cell characteristics, proliferation ability, and migration and invasion ability, suggesting that it may play an important role in GB progression.

### CA on proliferation, cancer stemness and metastatic capacity in GBM8401-CSCs cells

**Figure 2:**
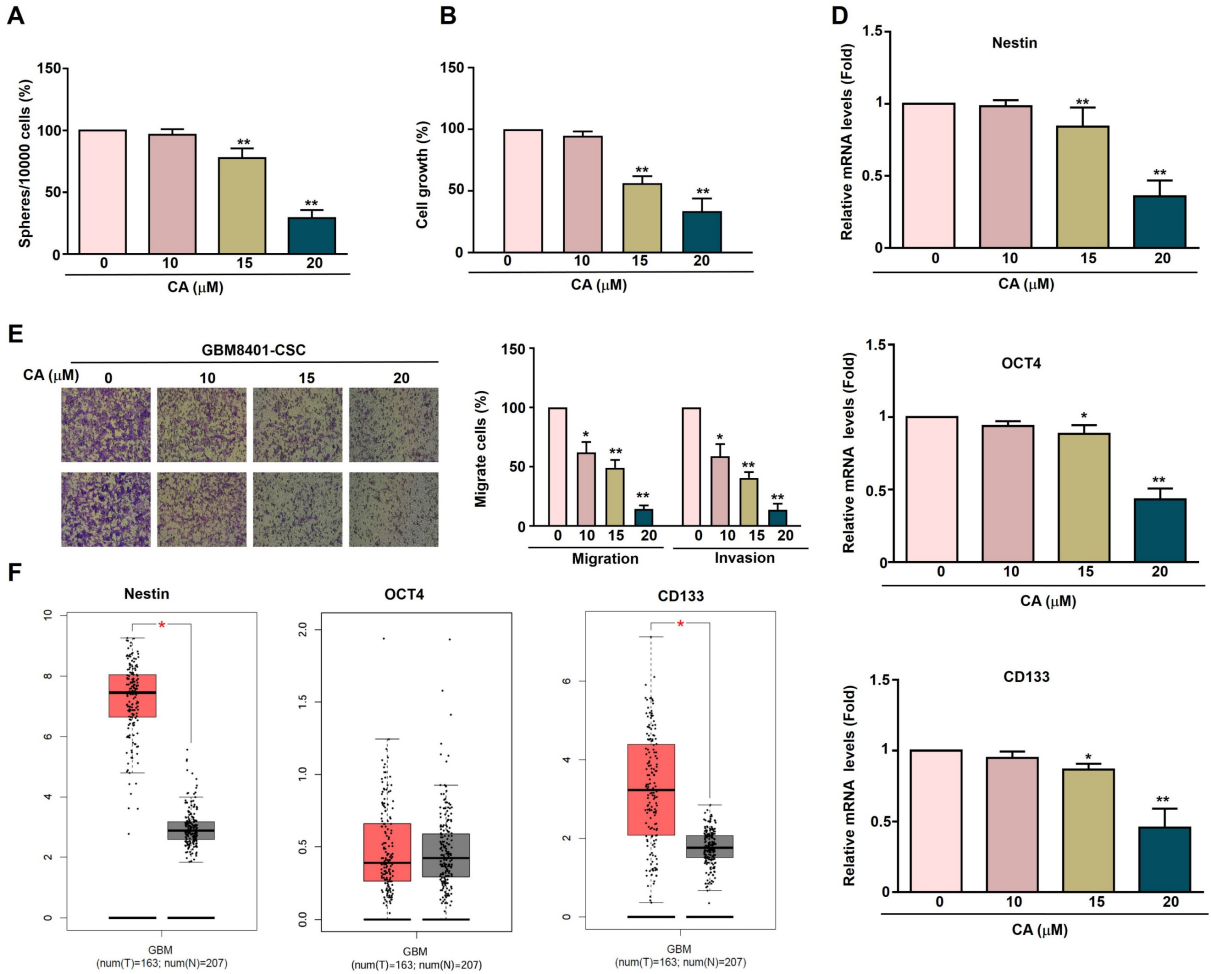
** Effect of CA on proliferation, sphere formation and metastatic capacity in GBM8401-CSCs.** (A) Human GBM8401-CSCs were treated with various concentrations of CA (0, 10, 15, and 20 μM) for five days, after which the cells were harvested to assess 3D sphere formation. (B) GBM8401-CSCs were treated with various concentrations of CA (0, 10, 15, and 20 μM) for 24 hours, and cell growth was assessed using the MTT assay. (C) Migration and invasion assays were performed on GBM8401-CSCs following 24 hours treatment with CA (0, 10, 15, and 20 μM). (D) The mRNA expression levels of the cancer stem cell markers Nestin, OCT4, and CD133 in GBM8401-CSCs were evaluated using RT-qPCR. (E) Relative mRNA expression levels of Nestin, OCT4, and CD133 in normal (n = 207) and tumor tissues (n = 163), based on data from the GEPIA database. *p < 0.05; **, *p*<0.01 indicates a significant difference.

To investigate the effect of CA on human GBM8401-CSCs, GBM8401-CSCs were exposed to increasing concentrations of CA (0, 10, 15, and 20 μM) for five days, followed by evaluation of 3D sphere formation. CA treatment significantly reduced the sphere formation ability of GBM8401-CSC (Figure 2A), indicating that it inhibits the self-renewal ability of tumor stem cells. To evaluate cell growth, GBM8401-CSCs were exposed to CA (0, 10, 15, and 20 μM) for 24 h, followed by the MTT assay. CA inhibited the growth of GBM8401-CSC in a dose-dependent manner (Figures 2B), showing its potential to reduce tumor proliferation. After CA treatment, the mRNA expression of the cancer stem cell-related genes Nestin, OCT4, and CD133 in GBM8401-CSC was significantly reduced (Figure 2D), further supporting the ability of CA to inhibit stem cell characteristics. The migration and invasion capability of human GBM8401-CSCs was evaluated after treatment with various concentrations of CA (0, 10, 15, and 20 μM) for 24 h. CA inhibited the migration and invasion of GBM8401-CSC in a dose-dependent manner (Figures 2E), showing its potential to resist glioblastoma cell metastasis. Relative mRNA expression levels of Nestin, OCT4, and CD133 in normal tissues (n = 207) and tumor tissues (n = 163) were obtained from the Gene Expression Profiling Interactive Analysis (GEPIA) database. Figure 2F shows that the expression levels of Nestin, OCT4, and CD133 in GBM tissues are significantly higher than those in normal brain tissues, suggesting that these markers are associated with the occurrence and development of glioblastoma.

### Synergistic effects of TMZ and CA on cell growth inhibition, apoptosis induction, and metastatic potential in GBM8401-CSCs

**Figure 3:**
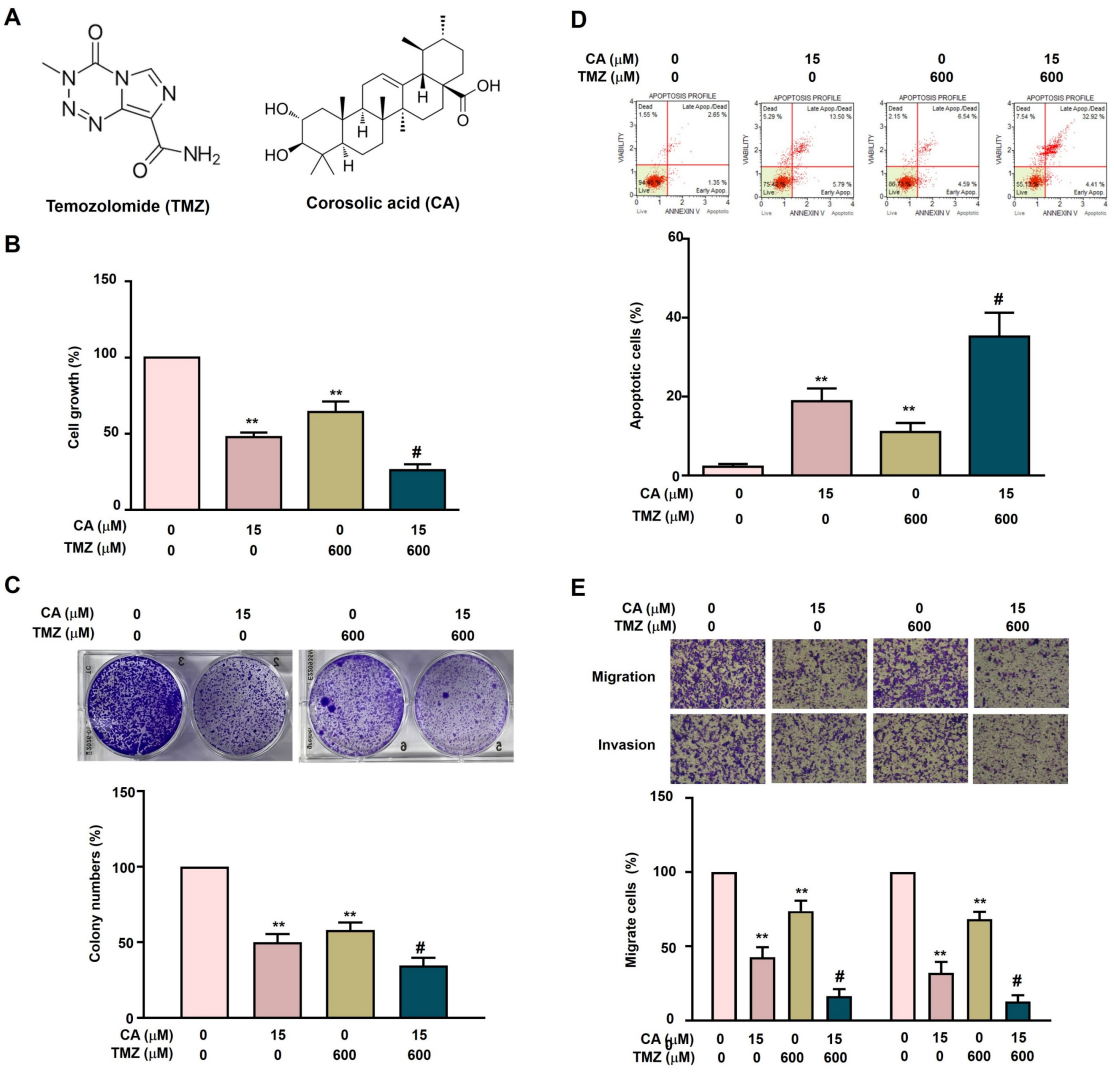
** Combined Effects of TMZ and CA on proliferation, apoptosis, and metastatic capacity in GBM8401-CSCs.** (A) Chemical structures of TMZ and CA. (B) Human GBM8401-CSCs were treated with various concentrations of TMZ (0 and 600 μM) and/or CA (0 and 15 μM), followed by evaluation of cell viability using the MTT assay, (C) colony formation ability, (D) apoptosis induction by flow cytometry, and (E) migration and invasion capacities. **p < 0.01 vs. control; #*p* < 0.05 vs. treatment with TMZ or CA alone.

The individual and combined effects of TMZ and CA on cell growth inhibition, apoptosis induction, and metastatic suppression were further investigated in GBM8401-CSCs. Cells were treated with TMZ (0 or 600 μM) and/or CA (0 or 15 μM), followed by assessment of cell growth and colony formation. Treatment with either TMZ (600 μM) or CA (15 μM) alone significantly inhibited cell growth and reduced colony formation. Notably, the combination of TMZ and CA exerted a greater synergistic inhibitory effect on both cell proliferation (Figure 3B) and colony formation (Figure 3C) than did either agent alone. In addition, treatment with either TMZ or CA alone significantly induced apoptosis (Figure 3D) and suppressed migration and invasion (Figure 3E) in GBM8401-CSCs. Importantly, combined treatment with TMZ and CA produced a markedly greater synergistic effect on apoptosis induction as well as on the inhibition of cell migration and invasion.

### Synergistic effects of TMZ and CA on cancer stemness activation in GBM8401-CSC cells

**Figure 4:**
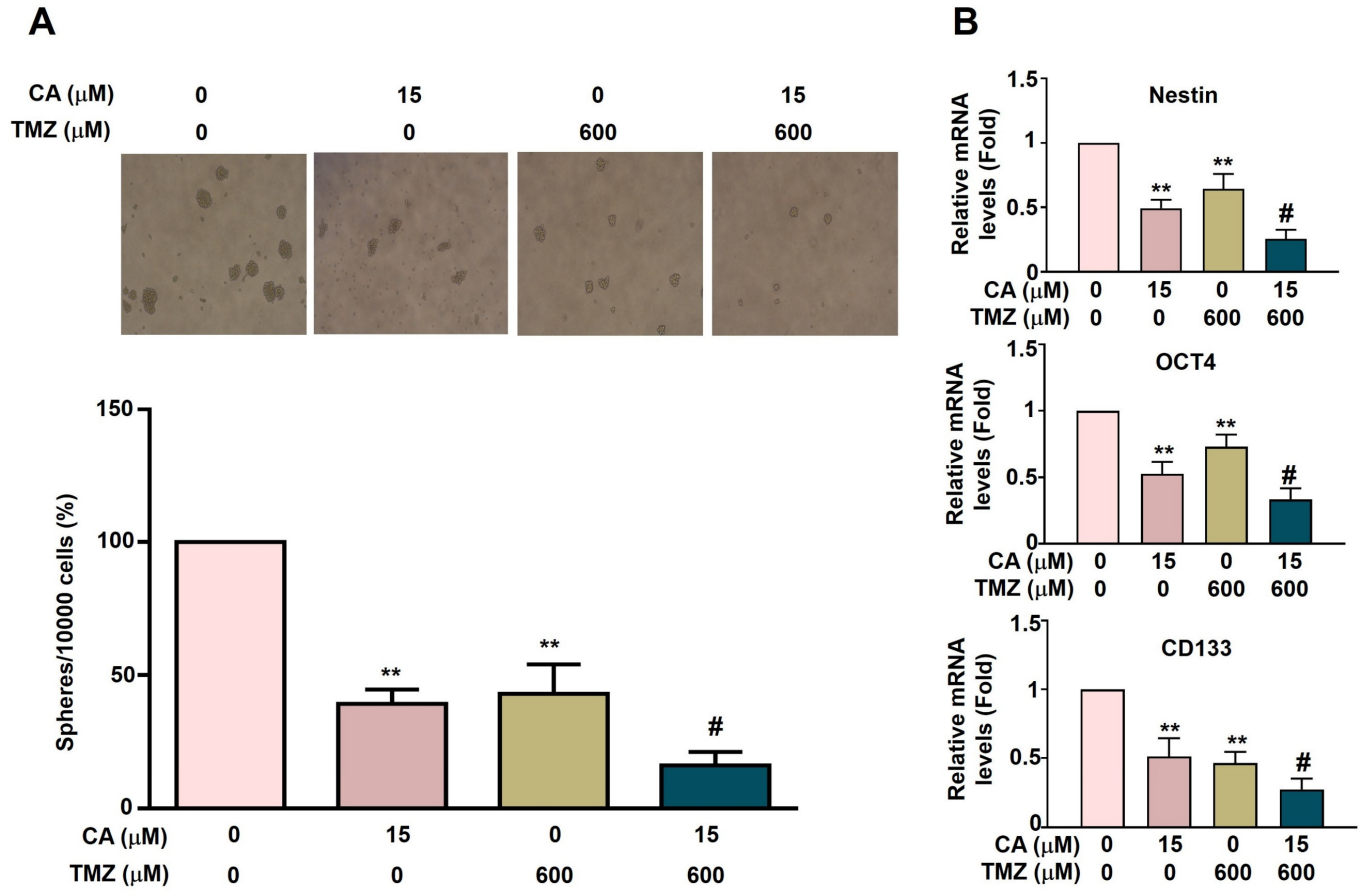
** Combined Effects of TMZ and CA on cancer stemness activation in GBM8401-CSCs.** (A) GBM8401-CSCs (10,000 cells) were treated with various concentrations of TMZ (0 and 600 μM) and/or CA (0 and 15 μM) for five days and then harvested to observe the 3D sphere formation, and (B) detect the expression of cancer stemness factors Nestin, OCT4, and CD133 via RT-qPCR assay. **p < 0.01 vs. control; #*p* < 0.05 vs. treatment with TMZ or CA alone.

To investigate the anti-cancer stemness effects of TMZ and CA in GBM8401-CSCs cells, the capacity for 3D sphere formation was assessed in human GBM8401-derived cancer stem-like cells (GBM8401-CSCs). Cells were treated with varying concentrations of TMZ (0 or 600 μM) and/or CA (0 or 15 μM) for five days. Both TMZ (600 μM) and CA (15 μM) alone significantly suppressed 3D sphere formation. Notably, combination treatment with TMZ and CA produced a greater synergistic inhibitory effect (Figure 4A). To further elucidate the mechanisms underlying this inhibition, the mRNA expression levels of key cancer stemness factors (Nestin, OCT4, and CD133) were analyzed. Treatment with either TMZ or CA alone markedly downregulated the expression of these factors. Importantly, a combination of TMZ and CA resulted in a more pronounced synergistic suppression of Nestin, OCT4, and CD133 expression (Figure 4B). These findings suggest that both TMZ and CA, particularly in combination, may exert therapeutic potential by impairing cancer stem cell characteristics, as evidenced by reduced sphere-forming ability and downregulation of stemness-associated genes.

### The potential binding affinity of TMZ and CA to cancer stemness factors CD133 and OCT4

**Figure 5:**
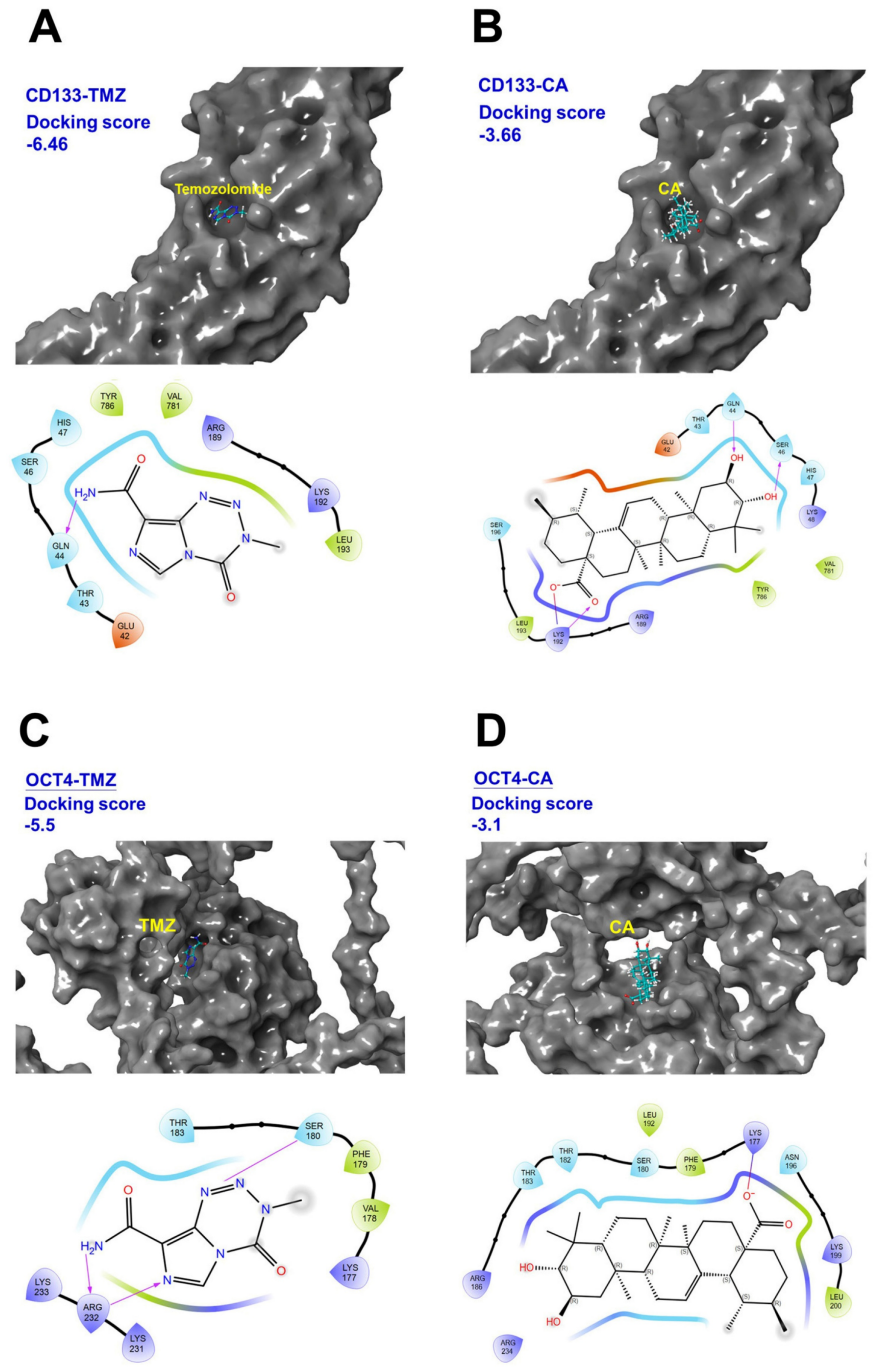
** Molecular docking of temozolomide (TMZ) and Corosolic acid (CA) with CD133 and OCT4 proteins.** (A) Structure of CD133-TMZ complex. (B) Structure of CD133-CA complex. (C) Structure of OCT4-TMZ complex. (D) Structure of OCT-CA complex. These images shows the results of molecular docking of TMZ and CA with CD133 and OCT4 proteins, and displays the corresponding docking scores and interaction maps.

The images in Figure 5 show the results of molecular docking of TMZ and CA with CD133 and OCT4 proteins and indicates the corresponding docking scores and interaction maps. A lower (more negative) docking score indicates a stronger binding affinity between the ligand and the target protein. As shown in Figures 5A-D, the docking score for the CD133-TMZ complex is -6.46, CD133-CA is -3.66, OCT4-TMZ is -5.50, and OCT4-CA is -3.10. These results suggest that TMZ exhibits the highest binding affinity toward CD133, whereas CA shows the weakest binding affinity to OCT4. The interaction map shows the atomic-level interactions between the ligand (TMZ or CA) and the protein binding site (e.g., hydrogen bonds, hydrophobic interactions), which helps to elucidate the binding mechanism (Figures 5A-D). These data can be used in the search and development of drugs to treat human GBM, including evaluating the ability of potential drug molecules to bind to target proteins, thereby providing a basis for designing more effective treatment strategies.

## Discussion

In this study, we found that GBM8401-CSCs exhibit increased self-renewal (3D sphere formation), proliferation, and colony-forming abilities. One of the most malignant features of GB is its capacity for infiltrative growth; GB cells can invade normal brain parenchyma and are often found several centimeters from the tumor core 23. Notably, the majority of tumor recurrences occur within 2 cm of the surgical resection margin, suggesting that these infiltrative GB-GSCs retain high tumorigenic potential 24. Our results also show that the combination of CA and TMZ has been shown to inhibit the proliferation and induce apoptosis in human GB-CSCs. Moreover, CA and TMZ co-treatment suppresses the migratory capacity of human GB-CSCs, potentially decreasing the likelihood of tumor spread and metastasis. CA combined with TMZ serves as a therapeutic strategy for targeting human GB-CSCs.

The identification and isolation of GB-CSCs is essential for advancing GB research and therapy 25. CD133, an early- and widely-studied marker, confers GB cells with increased tumorigenicity, self-renewal, and expression of stemness and drug resistance genes (e.g., BCRP1, MGMT), contributing to resistance against temozolomide, carboplatin, paclitaxel, and etoposide 26, 27. CD133 expression is elevated in recurrent tumors, correlating with treatment resistance and progression. These cells show strong sphere-forming and tumor-initiating capacity, confirming their GB-CSCs identity 28. GB-CSCs self-renewal is tightly regulated intrinsically by transcription factors SOX2, OCT4, and Nestin, which maintain stemness and inhibit differentiation 29, 30. OCT4, enriched in GB-CSCs and linked to poor prognosis, is stabilized by the deubiquitinase USP5 31. OCT4 also transcriptionally upregulates SOX2 via complex formation with SOX4, with palmitoylation strengthening this interaction and sustaining GB-CSCS stemness and tumorigenicity; thus, OCT4 palmitoylation is a novel therapeutic vulnerability 32, 33. Nestin, a cytoskeletal intermediate filament protein and GB-CSCs marker, promotes cell cycle progression and spindle assembly through interaction with βII-tubulin and regulates stemness, proliferation, and invasion via heat shock cognate 71 (HSC71), suggesting both as potential therapeutic targets 34. A Nestin⁺ GB-CSCs subset drives tumor recurrence after temozolomide treatment, and its selective ablation halts tumor growth, underscoring the role of GB-CSCs in therapy resistance and relapse 35. In this study, we found that CD133 and Nestin expression were significantly elevated in GB tissues compared to normal brain tissues. Additionally, GB-CSCs with higher expression levels of CD133, OCT4, and Nestin, exhibited greater sphere-forming, proliferative, migratory, and invasive capabilities.

The co-treatment of CA and TMZ significantly inhibited GB-CSCs proliferation and induced apoptosis, while also suppressing their migratory capacity. These findings suggest that the combination of CA and TMZ may reduce the risk of tumor spread and recurrence by targeting the GB-CSC population. Moreover, the therapeutic effect of CA in this context appears to be mediated, at least in part, through the downregulation of core stemness markers, including CD133, OCT4, and Nestin, all of which were significantly elevated in GB tumor tissues compared to normal brain tissues 36, 37. Notably, higher expression of CD133, OCT4, and Nestin was associated with enhanced self-renewal, proliferative, and invasive potential in GB-CSCs, further supporting their role in malignancy 37. In this study, we found that CA significantly inhibits the sphere-forming ability of GBM8401-CSCs, indicating reduced self-renewal, downregulates the expression of the stem cell markers Nestin, OCT4 and CD133, suggesting diminished stemness. Additionally, CA treatment impaired the migration and invasion of GBM8401-CSCs in a dose-dependent manner, demonstrating its potential to inhibit GB-CSCs metastasis. Another important point is that we did not suggest the antitumor effect of CA + TMZ in GB-CSCs using an *in vivo* animal model in the current study. In our future work, we will focus on *in vivo* studies using GBM8401-CSCs xenograft-bearing mice and GBM8401-CSCs orthotopic mouse models to further support and validate the antitumor effect of CA + TMZ, which warrants further investigation.

GB cells frequently develop both intrinsic and acquired resistance to TMZ, which significantly limits therapeutic efficacy and contributes to the short median survival observed in GB patients 38. Strategies to overcome TMZ resistance have focused on combining TMZ with sensitizing agents and targeting both tumor bulk and CSC populations 39. Our study highlights the potential of CA as a TMZ-sensitizing agent, particularly in its ability to target GB-CSCs. To further explore the molecular basis of CA and TMZ activity, we performed molecular docking analysis targeting CD133 and OCT4.the molecular docking of TMZ and CA with CD133 and OCT4 proteins revealed their respective docking scores: CD133-TMZ (-6.46), CD133-CA (-3.66), OCT4-TMZ (-5.50), and OCT4-CA (-3.10). These results suggest that both TMZ and CA exhibit notable binding affinity toward CD133 and OCT4. As a plant-derived compound with an established pharmacological safety profile, CA represents a promising natural, low-toxicity therapeutic candidate with potential for clinical translation. Within the framework of personalized medicine, CA may be incorporated into tailored treatment strategies, particularly for patients exhibiting resistance to conventional therapies. Taken together, these findings warrant further preclinical and clinical evaluation of CA as a novel, safe, and effective adjunct in the treatment of GB.

## Conclusion

Our results demonstrated that CA combined with TMZ individually inhibited cell proliferation and colony formation, induced apoptosis, suppressed migration and invasion, reduced 3D sphere formation, and downregulated the stemness markers Nestin, OCT4, and CD133, further inhibiting tumor growth, metastasis, and cancer stemness highlighting their potential as a combined therapy against GB-CSCs.

## References

[1] Darlix A, Zouaoui S, Rigau V, Bessaoud F, Figarella-Branger D, Mathieu-Daude H (2017). Epidemiology for primary brain tumors: a nationwide population-based study. J Neurooncol.

[2] Louis DN, Ohgaki H, Wiestler OD, Cavenee WK, Burger PC, Jouvet A (2007). The 2007 WHO classification of tumours of the central nervous system. Acta Neuropathol.

[3] Tykocki T, Eltayeb M (2018). Ten-year survival in glioblastoma. A systematic review. J Clin Neurosci.

[4] Louis DN, Perry A, Wesseling P, Brat DJ, Cree IA, Figarella-Branger D (2021). The 2021 WHO Classification of Tumors of the Central Nervous System: a summary. Neuro Oncol.

[5] Stupp R, Mason WP, van den Bent MJ, Weller M, Fisher B, Taphoorn MJ (2005). Radiotherapy plus concomitant and adjuvant temozolomide for glioblastoma. N Engl J Med.

[6] Sun T, Warrington NM, Luo J, Brooks MD, Dahiya S, Snyder SC (2014). Sexually dimorphic RB inactivation underlies mesenchymal glioblastoma prevalence in males. J Clin Invest.

[7] Molinaro AM, Taylor JW, Wiencke JK, Wrensch MR (2019). Genetic and molecular epidemiology of adult diffuse glioma. Nat Rev Neurol.

[8] Pouyan A, Ghorbanlo M, Eslami M, Jahanshahi M, Ziaei E, Salami A (2025). Glioblastoma multiforme: insights into pathogenesis, key signaling pathways, and therapeutic strategies. Mol Cancer.

[9] Reya T, Morrison SJ, Clarke MF, Weissman IL (2001). Stem cells, cancer, and cancer stem cells. Nature.

[10] Singh SK, Clarke ID, Terasaki M, Bonn VE, Hawkins C, Squire J (2003). Identification of a cancer stem cell in human brain tumors. Cancer Res.

[11] Diehn M, Cho RW, Clarke MF (2009). Therapeutic implications of the cancer stem cell hypothesis. Semin Radiat Oncol.

[12] Jin M, Li J, Zheng L, Huang M, Wu Y, Huang Q (2024). Corosolic acid delivered by exosomes from Eriobotrya japonica decreased pancreatic cancer cell proliferation and invasion by inducing SAT1-mediated ferroptosis. Int Immunopharmacol.

[13] Phakeovilay C, Bourgeade-Delmas S, Perio P, Valentin A, Chassagne F, Deharo E (2019). Antileishmanial Compounds Isolated from Psidium Guajava L. Using a Metabolomic Approach. Molecules.

[14] Zhang W, Men X, Lei P (2014). Review on anti-tumor effect of triterpene acid compounds. J Cancer Res Ther.

[15] Bahadori MB, Vandghanooni S, Dinparast L, Eskandani M, Ayatollahi SA, Ata A (2019). Triterpenoid corosolic acid attenuates HIF-1 stabilization upon cobalt (II) chloride-induced hypoxia in A549 human lung epithelial cancer cells. Fitoterapia.

[16] Fujiwara Y, Komohara Y, Ikeda T, Takeya M (2011). Corosolic acid inhibits glioblastoma cell proliferation by suppressing the activation of signal transducer and activator of transcription-3 and nuclear factor-kappa B in tumor cells and tumor-associated macrophages. Cancer Sci.

[17] Fujiwara Y, Komohara Y, Kudo R, Tsurushima K, Ohnishi K, Ikeda T (2011). Oleanolic acid inhibits macrophage differentiation into the M2 phenotype and glioblastoma cell proliferation by suppressing the activation of STAT3. Oncol Rep.

[18] Sun LW, Kao SH, Yang SF, Jhang SW, Lin YC, Chen CM (2021). Corosolic Acid Attenuates the Invasiveness of Glioblastoma Cells by Promoting CHIP-Mediated AXL Degradation and Inhibiting GAS6/AXL/JAK Axis. Cells.

[19] Yin H, Zhou Y, Wen C, Zhou C, Zhang W, Hu X (2014). Curcumin sensitizes glioblastoma to temozolomide by simultaneously generating ROS and disrupting AKT/mTOR signaling. Oncol Rep.

[20] Liu Y, Song X, Wu M, Wu J, Liu J (2020). Synergistic Effects of Resveratrol and Temozolomide Against Glioblastoma Cells: Underlying Mechanism and Therapeutic Implications. Cancer Manag Res.

[21] Chen PH, Lee CH, Liaw CC, Liang RT, Khan MAR, Tsai JN (2024). Metachromin C, a marine-derived natural compound, shows potential in antitumor activity. Int J Med Sci.

[22] Huang PY, Hung TW, Hsieh YH, Wu PJ, Chen PN, Lee CC (2025). Ellagic acid suppresses the human renal carcinoma cell migration and invasion by targeting the RUNX2/MMP1 expression. Int J Med Sci.

[23] Teodorczyk M, Martin-Villalba A (2010). Sensing invasion: cell surface receptors driving spreading of glioblastoma. J Cell Physiol.

[24] Winkler F, Kienast Y, Fuhrmann M, Von Baumgarten L, Burgold S, Mitteregger G (2009). Imaging glioma cell invasion *in vivo* reveals mechanisms of dissemination and peritumoral angiogenesis. Glia.

[25] Beier D, Hau P, Proescholdt M, Lohmeier A, Wischhusen J, Oefner PJ (2007). CD133(+) and CD133(-) glioblastoma-derived cancer stem cells show differential growth characteristics and molecular profiles. Cancer Res.

[26] Singh SK, Clarke ID, Hide T, Dirks PB (2004). Cancer stem cells in nervous system tumors. Oncogene.

[27] Liu G, Yuan X, Zeng Z, Tunici P, Ng H, Abdulkadir IR (2006). Analysis of gene expression and chemoresistance of CD133+ cancer stem cells in glioblastoma. Mol Cancer.

[28] Shin DH, Xuan S, Kim WY, Bae GU, Kim JS (2014). CD133 antibody-conjugated immunoliposomes encapsulating gemcitabine for targeting glioblastoma stem cells. J Mater Chem B.

[29] Hemmati HD, Nakano I, Lazareff JA, Masterman-Smith M, Geschwind DH, Bronner-Fraser M (2003). Cancerous stem cells can arise from pediatric brain tumors. Proc Natl Acad Sci U S A.

[30] Gangemi RM, Griffero F, Marubbi D, Perera M, Capra MC, Malatesta P (2009). SOX2 silencing in glioblastoma tumor-initiating cells causes stop of proliferation and loss of tumorigenicity. Stem Cells.

[31] Jiang X, You H, Niu Y, Ding Y, Chen Z, Wang H (2024). E2F1-regulated USP5 contributes to the tumorigenic capacity of glioma stem cells through the maintenance of OCT4 stability. Cancer Lett.

[32] Ikushima H, Todo T, Ino Y, Takahashi M, Saito N, Miyazawa K (2011). Glioma-initiating cells retain their tumorigenicity through integration of the Sox axis and Oct4 protein. J Biol Chem.

[33] Chen X, Niu W, Fan X, Yang H, Zhao C, Fan J (2023). Oct4A palmitoylation modulates tumorigenicity and stemness in human glioblastoma cells. Neuro Oncol.

[34] Wang Q, Wu H, Hu J, Fu H, Qu Y, Yang Y (2021). Nestin Is Required for Spindle Assembly and Cell-Cycle Progression in Glioblastoma Cells. Mol Cancer Res.

[35] Jiang Y, Zhou J, Luo P, Gao H, Ma Y, Chen YS (2018). Prosaposin promotes the proliferation and tumorigenesis of glioma through toll-like receptor 4 (TLR4)-mediated NF-kappaB signaling pathway. EBioMedicine.

[36] Selvaraj S, Srinivas BH, Verma SK, Ms G (2024). Significance of Nestin and CD133 as cancer stem cell markers in diffuse glioma and association with p53 expression and IDH status. Int J Clin Exp Pathol.

[37] Dai Y, Yu T, Yu C, Lu T, Zhou L, Cheng C (2022). ISG15 enhances glioma cell stemness by promoting Oct4 protein stability. Environ Toxicol.

[38] Sener UT, Sulman EP, Sarkaria JN (2024). Temozolomide use in elderly patients with MGMT promoter unmethylated glioblastoma: Is it finally time to dismount a dead horse?. Neuro Oncol.

[39] Jiapaer S, Furuta T, Tanaka S, Kitabayashi T, Nakada M (2018). Potential Strategies Overcoming the Temozolomide Resistance for Glioblastoma. Neurol Med Chir (Tokyo).

